# Corneal Epithelial and Vascular Tumors in Domestic Species: Narrative Review of the Literature and Insights from New Cases (2016–2025)

**DOI:** 10.3390/vetsci13030258

**Published:** 2026-03-11

**Authors:** Miriam Fossati, Gaia Beatrice Maria Bianchi, Chiara Giudice

**Affiliations:** Department of Veterinary Medicine and Animal Sciences, University of Milan (UNIMI), Lodi, 26900, Italy; miriam.fossati99@gmail.com (M.F.); gaiabeatrice.bianchi@unimi.it (G.B.M.B.)

**Keywords:** cornea, neoplasia, SCC, vascular tumor, hemangioma, hemangiosarcoma, papilloma, dog, cat, horse

## Abstract

Neoplasia of the cornea is overall rare, with corneal squamous-cell carcinoma (c-SCC) being most commonly reported in all species. C-SCC pathogenesis has been related to UV exposure in humans and horses, and to papillomavirus infection in humans. In dogs, brachycephalic conformation and chronic keratitis were associated with c-SCC. Corneal vascular tumors have also been exceptionally reported in humans, and rarely in animals. In dogs, they have been suggested to be UV-related. Except for equine c-SCCs, most studies on corneal neoplasms are case reports. The present study aimed to review the literature on epithelial and vascular corneal tumors in dogs, cats, and horses, adding new cases from our archives. Pubmed and Web of Science were searched (1980–2025) using the following keywords: cornea, neoplasia, carcinoma, hemangioma, hemangiosarcoma, dog, cat, and horse. Additionally, 94 new cases of corneal neoplasia were retrieved: 47 dogs (40 epithelial and seven vascular); 29 cats (14 epithelial and 15 vascular) and 18 horses. Signalment, clinical history, and histopathological characteristics were analyzed and compared with the literature. The combined results supported a strong association between brachycephalic dogs and c-SCC occurrence and highlighted the frequent coexistence in the feline species of symblepharon and corneal perforation, with corneal tumors.

## Simple Summary

Corneal neoplasms are rare in all species. Squamous-cell carcinoma and vascular tumors are the most frequently reported, with distinct etiopathogenesis across species. The present study reviews the current literature and describes 94 new cases (in dogs, cats, and horses) from our archives, aiming to contribute to current knowledge on corneal vascular and epithelial neoplasms in domestic animals. The combined results highlighted a strong association between brachycephalic dogs and c-SCC occurrence, and the frequent coexistence of symblepharon and corneal perforation with corneal tumors in cats.

## 1. Introduction

Primary corneo-limbal neoplasia are rare in domestic animals [1,2], as well as in humans [3].

In humans, carcinomas of the surface epithelium are the most common, and are collectively indicated as Ocular Surface Squamous Neoplasia (OSSN), referring to both conjunctival and corneal epithelial neoplasms [4]. Different factors have been implicated in their pathogenesis, e.g., ultraviolet (UV) light exposure, human papillomavirus (HPV), and immunodeficiency virus (HIV) infections [5,6]. Ocular papillomas have also been reported, frequently due to HPV infection and typically affecting only the conjunctiva (viral papillomas), although in adults papillomas extending to the cornea and predisposing to carcinoma have also been described [7]. Other primary corneal neoplasms have rarely been described: only single cases of corneal hemangioma, melanoma and myxoma have been reported [8,9,10].

Similar to humans, in dogs, cats, and horses, the most commonly reported corneal neoplasia is squamous-cell carcinoma (c-SCC), followed by vascular neoplasia [1,2].

The specific characteristics of c-SCC and other corneal neoplasms may vary greatly between species, possibly pointing to different pathogenetic mechanisms.

The objective of this study is to conduct a comprehensive review of cases of epithelial and vascular corneal tumors in domestic animals (dogs, cats, and horses), as documented in the literature and as retrieved from the archives of our institution, to investigate clinicopathological features that may provide insight into their pathogenesis.

## 2. Materials and Methods

For the present study, Pubmed and Web of Science were searched using the following keywords: cornea, neoplasia, carcinoma, hemangioma, hemangiosarcoma, dog, cat, and horse. Studies from 1980 to 2025 were considered. The bibliographies of the retrieved articles were also searched.

Data from the archives of Veterinary Anatomic Pathology of the Department of Veterinary Medicine and Animal Sciences (DIVAS), Università degli Studi di Milan, were reviewed.

From January 2016 to May 2025, a total of 4439 cases of ocular or periocular lesions were recorded. Cases of epithelial and vascular neoplasia originating primarily from the cornea or the corneal limbus in dogs, cats, and horses were selected, signalment and clinical history were examined, and histopathological glass slides and paraffin blocks were retrieved.

All samples were fixed in 10% buffered formalin and embedded in paraffin wax. From paraffin blocks, serial 5μ sections were cut and stained with hematoxylin and eosin.

All cases were re-evaluated by light microscopy, and the following parameters were recorded:

- Growth pattern (for epithelial neoplasia): In situ, exophytic, infiltrative, and stromal invasive. Tumors were defined in situ when growth was only intraepithelial, exophytic when the neoplasia formed a raised mass without infiltrating into the deeper stroma, infiltrative when extensively infiltrating into the stroma, and stromal invasive if neoplastic cells grew only into the stroma, forming solid islands surrounded by fibrous tissue with no apparent connection with the overlying corneal epithelium, which, in turn, had no sign of neoplastic transformation [11].
- Presence and degree of tumor-associated inflammation; semi-quantitative evaluation (0 = none, 1+ = mild, 2+ = moderate, and 3+ = severe).
- Presence of histopathological signs of UV exposure damage: Acantholytic cells and elastosis of the stromal collagen.
- Presence of cytopathic effects and inclusion bodies in neoplastic cells, suggestive of Papillomavirus infection.

Statistical analysis was performed on canine cases of c-SCC from our database by using GraphPad Prism 10.

## 3. Results

### 3.1. Literature Review

#### 3.1.1. Canine Corneo-Limbal Neoplasia

Epithelial neoplasia, namely, corneal c-SCC, is the most commonly reported corneo-limbal neoplasia in dogs [1,2]. C-SCC usually occurs in adult to senior dogs, between 6 and 14.5 years old. Brachycephalic breeds and dogs affected by chronic keratitis are more commonly affected. In a retrospective study [8] on 26 dogs with c-SCC, 21/26 were brachycephalic breeds. Moreover, all dogs had histological signs of chronic keratitis, with many also reporting a clinical diagnosis of keratoconjunctivitis sicca (KCS; 10/26), chronic superficial keratitis (CSK; 4/26), and pigmentary keratitis (4/26). Therefore, it has been suggested that chronic inflammation, related to both facial conformation and to specific corneal diseases, can predispose to c-SCC [12].

Another study, reporting three c-SCCs and one squamous papilloma, also registered a high prevalence of brachycephalic breeds (3/4) and chronic pigmentary keratitis (4/4) [13].

Other works reported single cases, for a total of seven cases, which, again, most frequently occurred in brachycephalic dogs (4/7) and/or dogs with either KCS or pigmentary keratitis (5/7) [14,15,16,17,18,19]. All studies are detailed in Table 1.

In the study by Dreyfus et al. [12], 16/26 dogs affected by keratitis had also undergone immunosuppressive therapy with either cyclosporine or tacrolimus; the authors, therefore, suggested that the role of immunomodulatory drugs in triggering the neoplastic transformation of corneal epithelial cells should be considered.

The role of UV exposure in the pathogenesis of c-SCC has also been investigated in different studies, mainly by immunohistochemical evaluation of p53 expression in neoplastic cells. The results, however, were inconsistent: six cases of c-SCC stained p53 positive [17,20], two cases were negative [19], and a case with actinic keratitis without neoplastic transformation stained positive [20].

**Table 1:** Corneal epithelial and vascular neoplasia in dogs.

Diagnosis	N° Cases	Site	Breed	Age (Years)	Clinical History	Ref.
SCC	1	Cornea	Golden Retriever	11	Trauma	[14]
SCC	1	Limbus	Shih Tzu	12	KCS	[15]
3 SCC + 1 papilloma	4	Cornea	Pug (2), Lhasa Apso, and Chow Chow	6–10.2	PK	[13]
SCC	1	Limbus	Border Collie	6	nr	[16]
SCC	1	Cornea	English Bulldog	6	KCS	[17]
SCC	2	Cornea	Pug, Toy Poodle	8–11	PK	[19]
SCC	1	Cornea	French Bouledogue	7	KCS and cyclosporine therapy	[18]
SCC	26	Cornea	Pug (9), Bulldog (5), Boxer (2), Pekingese (2), Cavalier King Charles Spaniel, Cocker Spaniel, Shih Tzu, Labrador crossbreed, Bassett Hound, Greyhound, Border Collie, and Chow Chow	6–14.5	10/26 KCS, 4/26 CSK, 4 PK, and 16/26 cyclosporine therapy	[12]
SCC	16 (14 dogs)	Cornea	Pug (6), Bulldog (4), Cavalier King Charles Spaniel (2), Jack Russel Terrier (1), and Golden Retriever	2.1–13.7	8/14 PK, 5/14 KCS, and 1/14 trauma	[21]
pSCC	1	Cornea	Golden Retriever	9	Cutaneous warts papillomavirus infection	[22]
Papilloma	1	Cornea	Maltese	5	KCS	[23]
Papilloma	1	Cornea	Beagle	10 mo	nr	[24]
HSA	1	Limbus	Border Collie	8	nr	[25]
HSA	1	Cornea	German Shepherd cross-breed	11	nr	[26]
HSA	1	Cornea	Mixed breed	9	nr	[27]
7 HSA + 7 HA	14	Cornea + limbus	Mixed breed, Tibetan terrier, German Shepherd (2), Shetland Sheepdog (2), English Setter, Border Collie, Labrador Retriever, Pitbull Terrier, Australian Shepherd (2), German Shorthaired Pointer, and Australian cattle dog	7–14	6/14 KCS, 1 phthisis bulbi, and 1 intraocular inflammation	[28]
HSA	1	Cornea	Boxer	10	nr	[29]

SCC, squamous-cell carcinoma; pSCC, pigmented squamous-cell carcinoma; HSA, hemangiosarcoma; HA, hemangioma; KCS, keratoconjunctivitis sicca; PK, pigmentary keratitis; CSK, chronic superficial keratitis; nr, not reported.

In a single case reported so far, a correlation between c-SCC and papillomavirus infection has been proven. A pigmented c-SCC was described in a 9-year-old Golden Retriever dog, with a previous clinical diagnosis of cutaneous papillomaviral warts at 8 months of age. Neoplastic cells of c-SCC showed typical papillomavirus-induced changes, and canine papillomavirus type 17 (CPV-17) was confirmed in the neoplastic tissue by PCR analysis [22].

Grossly, canine c-SCCs are mostly exophytic masses originating from the central cornea [12]. Histologically multiple growth patterns have been described, namely, intraepithelial (carcinoma in situ) [12,14]; exophytic, the most commonly diagnosed, with neoplastic cells invasion limited to the superficial corneal stroma [12,13,15,17,21]; combined exophytic and infiltrative, when the proliferative mass is associated with significant invasion of the underlaying corneal stroma [12,18,19,22]; and purely infiltrative, reported in only four cases, when the neoplasia infiltrates the corneal stroma, but no prominent surface growth can be identified [12,16]. A single case of a poorly differentiated c-SCC with a diffuse infiltrating growth pattern and pseudoacinar architecture has also been reported [18].

Thickening and hyperplasia of the corneal epithelium adjacent to the neoplasia are frequently reported, as well as stromal neovascularization and a variable degree of mixed inflammatory cell infiltration [16,17,19], consistent with a history of chronic keratitis.

Corneal squamous papillomas have only been described exceptionally in dogs, with two cases reported in the literature: two dogs were affected by squamous papilloma arising in chronic pigmentary keratitis or severe chronic KCS [13,23]. A single case of a 10-month-old Beagle, with multiple viral papillomas affecting the cornea, the conjunctiva and the eyelids has also been described [24].

Vascular neoplasia, hemangioma (HA) and hemangiosarcoma (HSA) are the second most common corneal tumors in dogs, although much rarer than c-SCC, with only 18 cases in the literature reported so far [25,26,27,28,29]. Vascular tumors originating from the cornea or the limbus of senior dogs, between 7 and 14 years old, and working dog breeds seem to be over-represented, suggesting that UV exposure can be a risk factor [30], although in only a few cases, this hypothesis could be supported by the finding of UV-related alterations in the surrounding cornea (i.e., elastosis), while most cases were associated with KCS [28]. Additionally, a few cases arose in dogs with a history of long-lasting topical immunosuppressive therapy with steroids, cyclosporine, or tacrolimus.

Macroscopically, irregular, raised, reddish, frequently bleeding masses, often associated with severe neovascularization and edema of the surrounding cornea, were described [28]. Microscopically, the corneal stroma is expanded by neoplastic endothelial cells arranged in trabeculae or in vascular/lacunar structures of varying sizes containing erythrocytes [26].

All studies are detailed in Table 1.

#### 3.1.2. Feline Corneo-Limbal Neoplasia

Primary corneal neoplasms in cats are rarer than in dogs. More commonly, the cornea is involved by the extension of intraocular or periorbital tumors, including eyelid SCC, uveal melanoma, lymphoma, and sarcoma [31].

Only seven cases of c-SCC have been reported so far in the feline species, three of which (3/7) extended to the limbus. In 4/7 cases, the tumor had a severely infiltrative growth pattern, extending into the anterior chamber in two cases [32,33,34]. In three c-SCC cases (3/7), co-occurrent vascular neoplasia (two HSA and one HA) was also observed, forming either a single or two separate masses [28,35,36].

Besides the three cases mentioned that occurred along with c-SCC, six additional cases of isolated corneal vascular neoplasms have been reported in cats [28,37,38].

Almost all the cases of either epithelial and/or vascular neoplasia occurred in cats with a clinical history of chronic inflammatory disease (see Table 2): chronic keratitis (7/13), chronic conjunctivitis (1/13), feline herpesvirus (FHV) keratitis (1/13), chronic upper respiratory problems associated with ocular discharge (1/13), episcleritis (1/13), and ocular trauma (1/13).

One study investigating signs of UV damage found no elastosis [28].

All studies are detailed in Table 2.

#### 3.1.3. Equine Corneo-Limbal Neoplasia

The most common corneo-limbal neoplasm, in horses, is squamous-cell carcinoma (c-SCC), which primarily affects adult or elderly horses, between 9 and 27.5 years of age [11].

Several predisposing factors have been identified for equine c-SCC. In an epidemiological study on ocular and adnexal SCCs in horses [39], the prevalence—recorded at 14 different American veterinary teaching hospitals—was higher in areas with greater annual solar exposure, higher longitude and altitude, and lower latitude. The same study retrospectively analyzed data from 147 cases of ocular SCC in horses and found a higher prevalence with increasing age and in geldings compared with intact males and females, as well as a predisposition in Appaloosas and draft breeds (Belgian, Clydesdale, and Shire) compared with Quarter Horses. Furthermore, horses with bay or black coats had a lower prevalence of SCC than those with other coat colors.

Haflingers are also over-represented in the equine c-SCC-affected population. Over a five-year period, 26% of c-SCC cases at the University of California–Davis Veterinary Hospital occurred in Haflinger horses (compared with less than 1% of all admissions during the same period). Analysis of the pedigree suggested a probable genetic predisposition with simple autosomal recessive inheritance [40]. A genome-wide association study identified a 1.5 Mb locus on chromosome ECA12 significantly associated with limbal SCC in Haflingers; within this locus, a missense mutation (c.1013 C > T p.Thr338Met) was found in the gene encoding for the damage-specific DNA-binding protein 2 (DDB2), which is involved in DNA repair processes through binding to DNA regions damaged by ultraviolet radiation [41]. The same and subsequent studies showed that homozygosity T/T for DDB2, compared with T/C and C/C genotypes, is an important risk factor for the development of limbal SCC in Haflinger, Belgian, Connemara, and Rocky Mountain Horse breeds, and reported the frequency of the T allele in various breeds: the highest frequencies were found in Haflinger (0.25), Belgian (0.21), Connemara (0.21), Rocky Mountain Horse (0.20), and Percheron (0.07) [41,42,43,44].

In some geographic areas, there is a marked prevalence of ocular SCC in Paint horses. At the University of Illinois, this breed accounted for 36% of SCC cases, suggesting a predisposition related to the lack of pigmentation of periocular tissues. Diluted coat colors such as Perlino and Cremello (homozygosis Cr/Cr at the MATP gene) and gray horses (STX17 gene) are also hypothesized to be risk factors, although further studies are needed [44]. Similarly, in a study on the treatment of limbal SCCs, the most represented breeds were Appaloosa and Paint [45].

A significantly higher expression of COX-1 and COX-2, compared with non-neoplastic cornea, has also been demonstrated in equine corneal SCC, whereas eyelids and nictitating membrane SCCs do not show significant differences in expression [46]. Other studies confirmed an increased COX-2 expression in ocular SCCs, but in a very low percentage of positive neoplastic cells, and the authors hypothesized that inflammatory rather than neoplastic cells were positive [47].

Typically, equine c-SCCs have a nodular, raised, whitish-pink appearance [48], but, not uncommonly, they grow infiltrating the corneal stroma and, rarely, the anterior chamber [49]; recurrence after incomplete resection has been reported [49,50]. Notably, a higher recurrence rate of limbal and bulbar conjunctival SCCs has been reported in the Thoroughbred breed and bay coat color, regardless of treatment [51].

A variant of c-SCC described in horses is defined as “corneal stromal invasive SCC (CSI-SCC)”. The tumor is characterized by extensive intrastromal growth while leaving the surface corneal epithelium intact. In one study, 23/87 (26%) ocular and periocular SCCs affected the cornea or the limbus, and 10/23 were diagnosed with CSI-SCCs.

In CSI-SCC cases, the cornea was thickened, edematous, with neovascularization, while neoplastic cells infiltrated the stroma, forming irregular cords surrounded by reactive fibrovascular tissue [11].

Corneo-limbal vascular neoplasia (HA and HSA) is uncommonly diagnosed in horses. One limbal HA [52], four limbal HSAs [53,54], and three corneal HSAs [28] have been reported so far. Vascular neoplasms predominantly affect adult horses between 9 and 21 years of age [28].

Macroscopically, they are raised, reddish masses, while microscopically, they show an architecture ranging from cavernous to mixed solid–cavernous, and moderate to severe associated lymphoplasmacytic inflammation [54].

In one study, all cases of HSA (3/3) were associated with elastosis [54], whereas in another study, elastosis was present in only 1/3 cases [28].

All studies are listed in Table 3.

### 3.2. Personal Data

#### 3.2.1. Canine Corneo-Limbal Neoplasia

In our cohort, 206 canine corneo-limbal lesions out of 3015 canine ocular cases were recorded; of these, 44 were epithelial neoplasia (44/206). Four cases were either recurrences or second biopsies following an incomplete first excision, with the second sample maintaining the same histopathological characteristics as the first, and one dog had a bilateral lesion; therefore, a total of 40 dogs were affected, and 41 neoplasia were counted. All data are listed in Table A1 and Table A2 in the Appendix A.

The mean age was 9.4 years, ranging from 4 to 15 years. No difference between sexes was observed: 20 dogs were males (16 intact and four neutered), 17 females (five intact and 12 spayed), and in three cases, the sex was not reported.

Brachycephalic breeds were over-represented (33/40 dogs; 82.5%). The most common were Pugs (8/40; 20%) and English Bulldogs (10/40; 25%), followed by Shih Tzu (7/40; 17.5%), mixed breeds (5/40; 12.5%), French Bouledogue (3/40; 7.5%), and Cavalier King Charles Spaniels (2/40; 5%). Singly represented were a Chihuahua, a Boston Terrier, a Pekingese, a Golden Retriever, and a German Shepherd cross.

When data were statistically examined, among dogs with ocular lesions, brachycephalic breeds had a markedly increased risk of epithelial corneal neoplasia compared with non-brachycephalic breeds (RR = 16.9; 95% CI: 7.5–38.1; *p* < 0.0001; power > 99%). Within the same population, brachycephalic breeds were also at significantly higher risk of developing corneo-limbal lesions (RR = 3.62; 95% CI: 2.77–4.73; *p* < 0.0001; power > 99%). When the analysis was restricted to dogs affected by corneo-limbal lesions, brachycephalic breeds remained at increased risk of epithelial corneal neoplasia (RR = 4.66; 95% CI: 2.16–10.1; *p* < 0.0001; power > 99%).

In all cases for which clinical history was available (26/40), chronic keratitis was reported. Twenty-one cases (21/26) were diagnosed with KCS, of which 12/21 received immunosuppressive therapy with either cyclosporine or tacrolimus. Five (5/26) cases were affected by undefined keratitis (3/5), 1/5 had bilateral keratoconjunctivitis and panophthalmitis due to Leishmania spp. infection, and 1/5 had a conjunctival flap for an unspecified ocular surgery.

**Figure 1:**
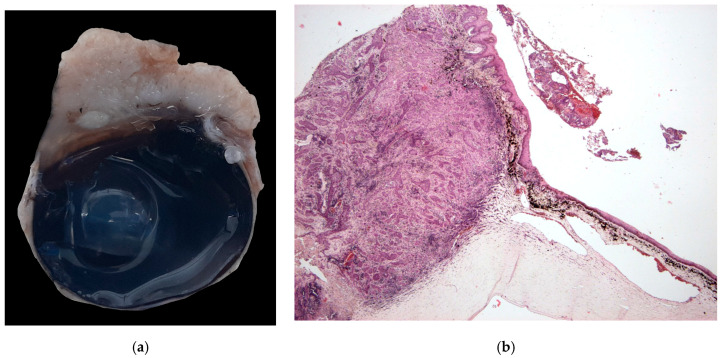
(**a**) Dog, eye globe, macroscopic picture of cross-section: a large exophytic c-SCC affects the entire surface of the cornea, extending focally to the limbus; (**b**) dog, eye globe: histological subgross view of a typical exophytic c-SCC, not infiltrating the deeper corneal stroma (EE, 4×).

**Figure 2:**
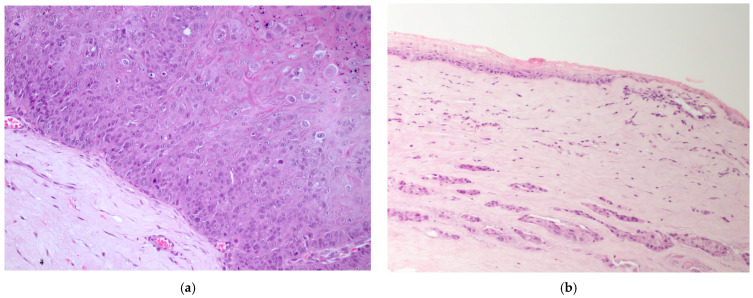
(**a**) Dog, corneal biopsy: corneo-limbal papilloma with scattered koilocytes, suggesting a viral origin (EE, 10×); (**b**) dog, corneal biopsy: neoplastic cells grow into the corneal stroma apparently without connection with the surface epithelium (stromal invasive pattern) (EE, 10×).

The majority of epithelial neoplasia were SCC (38/41), 2/41 were papillomas, one of which had focal transformation into in situ SCC. All c-SCC and papillomas grew in the central cornea, variably extending to the limbus (Figure 1a,b), except one papilloma with histopathological signs of viral infection, which primarily arose on the limbus (Figure 2a).

c-SCCs mostly grew as exophytic masses with limited infiltration into the corneal stroma (24/38). In only 8/38 cases, infiltrative growth into the stroma was pronounced; of these, 2/8 cases showed a “stromal invasive pattern”, forming islands of neoplastic cells that dissected the corneal stroma, while the overlaying corneal epithelium was apparently unaffected (Figure 2b). Six cases were in situ SCC (6/38). In one case, neoplastic cells were focally arranged in a pseudoacinar pattern.

Moderate or severe inflammation was present in more than half of the cases (23/41), while the others showed mild (12/41) or no inflammation (6/41). Pigmentary keratitis was histologically evident in cases clinically affected by KCS.

Elastosis was never observed, while acantholytic cells were present in 15/41 cases.

Seven cases of vascular neoplasia were diagnosed out of the 206 canine corneo-limbal lesions (7/206), HSAs and 2 HAs. Most (6/7) had significant limbal involvement, while one HA grew in the central cornea. Inflammation was mild or absent in all cases, and elastosis was observed in the adjacent bulbar conjunctiva in 3/7 cases.

The mean age of affected dogs was 7.6 years, ranging from 5 to 11 years. All affected dogs were female (two intact and five neutered). Herding breeds were most represented with 2/7 Australian Shepherds, 1/7 Abruzzese–Maremmano Shepherd Dog cross, and 1/7 German Shepherd Dog cross.

In one single case (case 2, Table 1, Appendix A), a clinical history of KCS, treated with local cyclosporine and corticosteroids for 8 years, was reported. The same dog was diagnosed with corneal HSA on the contralateral eye 7 years before. In all other cases, the neoplasia was the presenting complaint with no previous clinical history of ocular diseases.

#### 3.2.2. Feline Corneo-Limbal Neoplasia

In the present cohort, feline corneo-limbal lesions accounted for 104 cases out of 1042 ocular lesions; 17/104 were epithelial neoplasia. One cat (1/17) had an SCC in one eye and an HSA in the other. All data are listed in Table A3 and Table A4 in the Appendix A.

Three of the 17 epithelial cases were either recurrences or enucleations following the first biopsy, with a total of 14 affected cats, and 14 neoplasia were, therefore, considered.

Squamous-cell carcinoma was diagnosed in all cases (14/14). The mean age of affected cats was 8.7 years, ranging from 4 to 18 years. Most cats were males (8/14; 57%, one intact and seven neutered), and 4/14 (29%) were females (two intact and two spayed). Sex was not reported in two cases. All cats were European Domestic Short Haired.

Clinical history was available in seven cases: five cats were affected by symblepharon, with concurrent FHV1 infection and chronic keratitis in 2/5 cases. In 2/7 cases, a previous ocular trauma and eosinophilic keratitis were reported, respectively.

Five (5/14) tumors were limited to the cornea, while 9/14 extended to the limbus. In the majority of cases (10/14), the neoplasms had an infiltrative growth within the corneal stroma; 3/10 had a stromal invasive pattern of growth; and 3/10 had focal pseudoacinar formations. Four cases (4/14) showed a predominantly exophytic growth with minimal invasion into the underlying stroma.

Inflammation was mostly moderate (6/14) and mild or absent (5/14), while severe inflammatory cell infiltration was observed in three cases (3/14). Elastosis was observed in peritumoral tissues in one single cat whose contralateral eye was affected by HA (case 13, Table A3, Appendix A).

**Figure 3:**
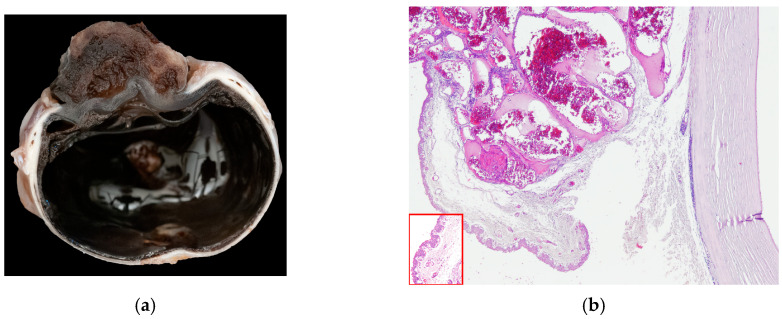
(**a**) Cat, eye globe, a large vascular neoplasm grows onto the corneal surface; (**b**) cat, eye globe: corneal hemangioma and symblepharon; conjunctival tissue relies on corneal stroma, substituting corneal epithelium, with tumor expanding its lamina propria (EE, 4×). Inset: Detail of the conjunctival epithelium and stroma that substitutes superficial corneal layers (EE, 40×).

In 15 cats (15/104), a vascular corneal neoplasia was diagnosed: 8/15 were HAs and 7/15 were HSAs (Figure 3).

The mean age of affected cats was 9.6 years, ranging from 3 to 14 years. Males were more represented (10/15; 67%; three intact and seven neutered) than females (5/15; 33%; two intact and three neutered). All cats were European Domestic Short Haired.

There was a history of symblepharon and/or FHV1 infection in 6/15 cases, traumatic injuries such as corneal ulcer or perforation in 2/15 cases, and 1/15 congenital palpebral malformation. The clinical history was not available in 6/15 cases.

Fourteen (14/15) neoplasia grew onto the cornea; in one single case (1/15), there was limbal extension. Inflammation was mild or absent in most cases (10/14) and moderate in the others (4/14). In one single case, stromal elastosis was observed (case 14, Table A4, Appendix A).

#### 3.2.3. Equine Corneo-Limbal Neoplasia

Two hundred corneo-limbal lesions out of 292 equine ocular samples were recorded; 19/200 were neoplastic, all of which were SCCs. One was a bilateral lesion, and one was a recurrence; therefore, 18 horses were affected.

All data are listed in Table A5 in the Appendix A.

The mean age of affected horses was 17.8 years, ranging from 9 to 29 years. There were 10/18 mares and 8/18 geldings.

The reported breeds were distributed as follows: Argentine Criollo (3/18), Arabian (2/18), Appaloosa (2/18), Haflinger (1/18), Haflinger–Appaloosa cross (1/18), Paint Horse (1/18), Mérens (1/18), Maremmano (1/18), KWPN (1/18), Welsh Pony (1/18), and not-specified pony (1/18). In three cases, the breed was undetermined (3/18).

**Figure 4:**
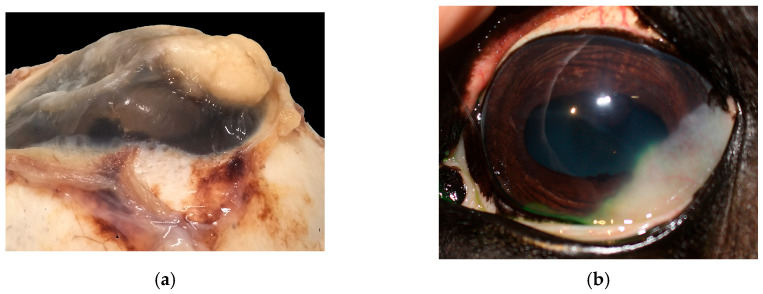
(**a**) Horse, formalin-fixed eye globe, gross features of corneo-limbal SCC (courtesy of Dr. Riccardo Stoppini); (**b**) horse, eye, clinical aspect of a stromal invasive SCC (courtesy of Dr. Samuela Mazzucchelli).

**Figure 5:**
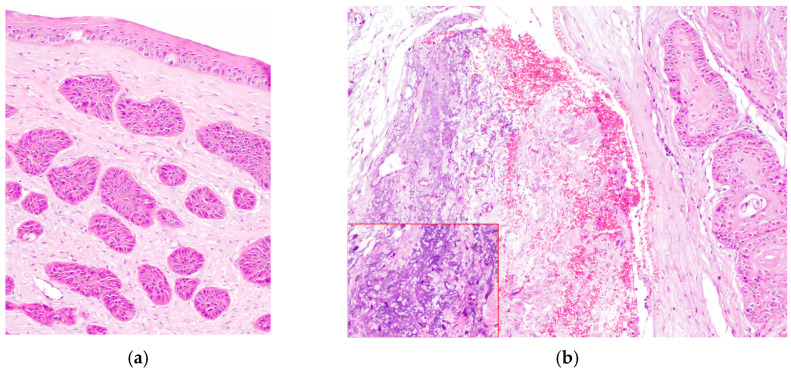
(**a**) Horse, cornea: typical features of stromal invasive SCC; neoplastic cells form islands deep in the stroma, without connection with the corneal epithelium (EE, 10×). (**b**) Horse, limbus: infiltrative corneo-limbal SCC; severe elastosis is recognizable in the conjunctival lamina propria (EE, 4×). Inset: Detail of the conjunctival elastosis (EE, 40×).

In the majority of cases, the tumor extended to the limbus (14/19) (Figure 4a), while fewer were limited to the cornea (5/19). Most neoplasms grew infiltrating the stroma (10/19), two of which (2/10) showed a stromal invasive pattern (Figure 4a and Figure 5a). Six SCCs (6/19) were exophytic, and three (3/19) were in situ SCC.

The degree of inflammation was equally distributed between moderate and severe (10/19 cases) and mild or absent (9/19 cases). Elastosis of the bulbar conjunctiva (Figure 5b) and acantholytic cells were evident in 9/19 cases.

## 4. Discussion

Primary corneal neoplasia are rare in humans and domestic species, with tumors of epithelial origin being the most frequently reported [1,2,3,31].

The incidence of human ocular surface squamous neoplasia (OSSN) has been calculated at 0.01 to 3.4 per 100,000 people/year. The pathogenesis of human OSSN has been associated with UV-B radiation and HPV infection, which can damage the p53 tumor suppressor gene and promote cellular replication, respectively. Secondary factors that have been involved in the oncogenesis of OSSN by causing immunosuppression are HIV infection, solar exposure (in particular, UV-A radiation), and vitamin A deficiency [6]. OSSN more frequently originates from the nasal limbus and expands onto the cornea or conjunctiva. A single case of SCC arising from the central cornea has been reported in a man affected by pannus keratitis and who had previously undergone cataract surgery [57].

Tumors of endothelial vascular origin are the second most common in domestic animals such as dogs, cats, and horses, while a single case of corneal hemangioma has been reported so far in humans [8].

### 4.1. Canine Corneo-Limbal Neoplasia

In dogs, c-SCCs more commonly originate in the central cornea, rarely extending to the limbus: 51/53 cases, reported in the literature between 1987 and 2025 [12,13,14,15,16,17,18,19,21,22], and 38/38 cases in the present cohort had a central corneal origin.

A strong relation between corneal SCC, brachycephalic breed, and a clinical history of chronic keratitis has been observed in dogs.

Brachycephalic breeds, in particular Pugs and Bulldogs, were overrepresented in many studies [12,13,15,17,18,19,21], and accounted for 82.5% of our cases, with Pugs, English Bulldog, and Shih Tzu being the most represented in our cohort.

Although referring to the general population was not possible, the statistical analysis of data in our population strongly confirmed the brachycephalic predisposition suggested by the literature, both regarding corneo-limbal vascular neoplasia and corneo-limbal lesions, with a 16.9-fold and 3.62-fold increased risk, respectively. It seems conceivable that, rather than a true predisposition, this phenomenon reflects the greater frequency, in these breeds, of corneal diseases, possibly also related to their facial conformation. Stratifying the analysis by corneo-limbal lesions suggests that these lesions may partially mediate the increased risk of epithelial corneal neoplasia in brachycephalic breeds, as the effect size was attenuated (a 4.66-fold increased risk) compared with the overall ocular lesion population (16.9).

Brachycephalic dogs are also predisposed to chronic keratitis, such as KCS or pigmentary keratitis, which have been reported to coexist with c-SCC in the majority of the previously cited cases [12,13,15,17,18,19,21] and in our cohort.

Chronic inflammation, with persistent activation of the inflammatory cascade, increasing cell proliferation and neoangiogenesis, has been previously implicated in the pathogenesis of ocular SCC [12,58].

Additionally, dogs affected by KCS frequently have a history of long-lasting local immunosuppressive therapy [12,18], and 12/21 dogs in our cohort for which clinical history was available had been treated with cyclosporine or tacrolimus. Immunosuppressive therapy has been proposed to play a role in c-SCC pathogenesis [12]; however, immunosuppressive drugs are largely used in canine ophthalmology, while corneal SCCs are comparatively rare. Therefore, larger epidemiological studies would be necessary to support this hypothesis.

Unlike humans, the role of UV exposure in the pathogenesis of canine c-SCC is still controversial, with studies of p53 reporting inconclusive results [17,19,20]. In the present cohort of cases, the presence of elastosis and acantholysis, possible histological signs of damage from solar exposure, was evaluated; no case showed elastosis, and scattered acantholytic cells were rarely observed, whose presence was not enough to confirm a solar-induced pathogenesis.

Corneal papillomas have been rarely reported, with three cases in the previous literature and three cases observed in the present study [13,23,24], and are most frequently classified as idiopathic, i.e., with no evidence of papillomavirus infection. Canine papillomavirus (CPV) infection can rarely result in the development of corneo-limbal viral papillomas, as reported in one of our cases and one literature case, which self-resolved after a few months [24], but in light of one SCC case with confirmed CPV infection [22], its role in the development of corneal SCC cannot be excluded, although further molecular and immunohistochemical studies are needed.

Most reported canine c-SCCs are either in situ SCCs [12,14] or raised exophytic masses on the corneal surface, uncommonly infiltrating the deeper stroma [12,18,19,21], and rarely infiltrating the stroma without being associated with a raised mass [12,16]. Most of our cases were exophytic (24/38), and fewer were in situ SCCs (6/38). A minority of cases showed an infiltrative growth pattern (8/38) that in two cases (2/8) was associated with an exophytic lesion. In two cases (2/8), the tumor was characterized by a distinctive intrastromal growth, while the surface corneal epithelium was apparently intact, similar to what has been described in horses as stromal invasive SCC [11]. Although not reported before, it is the authors’ opinion that canine c-SCC, although less frequent than in other species, can also be characterized by a stromal invasive growth pattern.

Corneal vascular neoplasia is overall rare in dogs, with only 18 cases reported in the literature so far [25,26,27,28,29]. Seven additional cases have been observed by the authors, with a slightly younger mean age of affected dogs (7.6 years) than in previously reported cases (9.9 years).

Female dogs were overrepresented in our cohort (7/7), while a difference in sex distribution was not observed in the literature. Because the data are limited, further studies are needed to determine if there is a real predisposition of female dogs to corneal vascular tumors.

Working breeds are overrepresented in the literature [25,26,28] and in the present cohort of cases, among dogs affected by both corneal and conjunctival [30] vascular neoplasia. Since these dogs are expected to have an outdoor lifestyle with greater exposure to ultraviolet (UV) radiation, it has been hypothesized that this breed distribution could support a pathogenetic role of UV exposure. However, in the authors’ opinion, this observation is insufficient to confirm a role of UV exposure in the pathogenesis of vascular corneal tumors, since we lack any information about the actual lifestyle of affected dogs.

Signs of elastosis were recorded in only 2/14 cases in one study [28], while most cases had previous KCS (6/14) or other ocular lesions (2/14), and histopathological signs of elastosis were observed in three (3/7) of our cases, only one of which was a sheepdog. Once again, since most tumors were excised with minimal surrounding normal tissue, these findings should be interpreted cautiously.

### 4.2. Feline Corneo-Limbal Neoplasia

In the feline species, corneal neoplasms are overall rarer than in dogs, with four reported cases of c-SCC, six of vascular neoplasia and three of co-occurring epithelial and vascular neoplasia [28,32,33,34,35,36,37,38]. The present caseload added a further 14 c-SCC and 15 vascular tumors (8/15 HA and 7/15 HAS).

A clinical history of chronic keratitis or conjunctivitis consistent with FHV infection or ocular trauma is frequently reported in the literature in cats with c-SCC, as well as in the present cohort of cases, where symblepharon, possibly related to previous FHV infection, was reported in five cases.

Feline c-SCC most commonly affected the limbus (9/14), both in the present cohort of cases and consistently with previous literature [32,35], contrary to canine and similarly to humans’ cases. Moreover, feline c-SCC were characterized by an infiltrative growth pattern within the corneal stroma, rather than forming an exophytic mass [32,33,34]. In our cases, 10/14 grew mainly infiltrating in the stroma, of which three (3/10) had a stromal invasive pattern.

Previous corneal lesions, traumatic or inflammatory, were also reported in most cases of vascular neoplasia and most likely played a significant role in its pathogenesis, being responsible for profound corneal stroma modification associated with vessel proliferation in or onto the normally avascular cornea.

### 4.3. Equine Corneo-Limbal Neoplasia

Corneo-limbal SCC is more common in horses than in dogs and cats [31], and many studies investigated its epidemiology, histopathology, possible pathogenetic factors, and treatments, often addressing not only corneo-limbal cases but ocular surface and ocular adnexa neoplasia as a whole.

Compared with the previous literature, the present caseload is limited, consisting of only 19 corneo-limbal SCCs. The mean age of our cases was 17.8 years, only slightly older than the mean age of 16.7 years reported in the literature [11]. Some studies reported a higher percentage of male horses, both stallions and geldings, compared with females, around 60–80% of their cases [11,40,45], but no difference was evident in our cases (10/18 mares and 8/18 geldings).

Multiple studies have confirmed a genetic predisposition for these neoplasms in some equine breeds, i.e., Haflinger, Belgian Draft, Connemara, Rocky Mountain, and Percheron, with a mutation of the DDB2 gene, which is involved in DNA repair processes after sun exposure damage [41,42,43,44]. While most of these breeds are uncommon in our country, the Haflinger is widespread; nevertheless, among our cases, only one Haflinger and one Haflinger–Appaloosa cross was present. A study reported that many affected Haflinger horses shared a common ancestor from the A bloodline, which is the most represented in the USA [40], while the N bloodline is more widespread in the other countries, and in Italy, all seven bloodlines are present. However, the limited number of our cases could determine a bias in breed distribution, so further studies with a higher sample size and investigating the prevalence of the mutated DDB2 gene are necessary.

Another predisposing factor for both corneo-limbal and conjunctival SCC in horses is sun exposure, analyzed as prevalence in different geographical areas [39]. Overrepresented breeds that do not share the DDB2 gene mutation, such as Appaloosa and Paint Horses [11,39,44,45,51,55,56], are characterized by multiple areas of non-pigmented skin, including the periocular tissues. Other genes that determine a reduced pigmentation of skin and/or haircoat, such as the cream (MATP) and gray (STX17) genes, have also been proposed as risk factors but are not yet confirmed [44]. One study also investigated the presence of elastosis as a histological hallmark of sun exposure damage and found 2/10 cases to be positive [11]. Elastosis and acantholytic cells were present in half the cases in the present caseload (9/19). It should also be noted that in the remaining cases, biopsies were represented almost entirely by neoplastic tissue, with insufficient surrounding tissue to investigate the presence of elastosis.

As stated before, equine SCCs have a predominantly corneo-limbal origin, similar to humans and cats. They can grow as an exophytic mass [48], but also infiltrate the corneal stroma [49] and not rarely with the peculiar “stromal invasive” growth pattern that apparently spares the surface epithelium [11]. Consistent with the previous literature, more than half of the cases (10/19) in the present cohort had an infiltrative growth pattern, of which 2/10 were stromal invasive.

Corneo-limbal vascular neoplasia has been reported more rarely in horses than in other domestic species, with only eight cases in the literature [28,52,53,54]. No case of corneo-limbal HA or HSA was present in our database. The role of sun exposure has been investigated in two studies that found elastosis in a total of 4/6 cases [28,54].

## 5. Conclusions

Corneal neoplasia is overall rare in both human and domestic animals, with SCC and vascular tumors being most frequently reported. Although no definite conclusion can be drawn, both a thorough examination of the current literature and a review of personal cases pointed to different pathogenetic mechanisms in different species. While UV exposure and papillomavirus infection have been advocated as the major predisposing factors to c-SCC in humans and horses, in dogs, c-SCCs are strongly related to brachycephalic breeds and a history of chronic keratitis (mostly KCS).

In the feline species, the rarity of epithelial and vascular corneal neoplasia makes speculation on their origin difficult. In the present study, we reported a relatively high number of cats affected by epithelial and vascular corneal tumors in which a previous history of symblepharon, trauma, or iris prolapse and entrapment was reported. The profound alteration in corneal stroma and marked vascular proliferation associated with these diseases could play a role in feline corneal neoplasia pathogenesis.

## Figures and Tables

**Table 2:** Corneal epithelial and vascular neoplasia in cats.

Diagnosis	N° of Cases	Site	Breed	Age (Years)	Clinical History	Ref.
SCC	2	Limbus (+cornea and anterior chamber)	DSH	13–16	Chronic keratitis	[32]
SCC	1	Cornea	Norwegian Forest	15.5	FHV-associated keratitis	[33]
SCC	1	Cornea	DSH	9	Chronic conjunctivitis	[34]
SCC+ HA	1	Limbus	DSH	14	NR	[35]
SCC+ HSA	1	Cornea	DSH	14	Chronic respiratory problems and ocular discharge	[36]
1 HSA + SCC, 3 HSA, and 1 HA	5	3 cornea, 2 cornea + limbus	4 DSH and 1 DMH	7–10	3 chronic keratitis, 1 ocular trauma, and 1 episcleritis	[28]
HSA	1	Cornea	DSH	10	Chronic keratitis	[37]
HSA	1	Cornea	DMH	7	Corneal fibrosis and neovascularization	[38]

SCC, squamous-cell carcinoma; HSA, hemangiosarcoma; HA, hemangioma; DSH, Domestic Short Haired; DMH, Domestic Medium Haired; FHV, Feline Herpesvirus; NR, not reported.

**Table 3:** Corneal epithelial and vascular neoplasia in horses.

Diagnosis	N° Cases	Site	Breed	Age (Years)	Clinical History	Ref.
SCC	147	Eye and adnexa	Appaloosa, Belgian Draft, Clydesdale, and Shire	11.1 ± 0.39	NR	[39]
SCC	157	Eye and adnexa (30 cornea, and 28 limbus)	QH, Appaloosa, and Paint	12.2	NR	[51]
SCC	57	Eye and adnexa	13 Paint, 6 Haflinger, 6 QH, 5 Appaloosa, 4 Selle français, 3 Arabian, 2 French Trotter, and 12 other breeds	7–23	NR	[44]
SCC	4	Limbus	2 Appaloosa,1 Belgian, and 1 QH	6–13	NR	[55]
SCC	1	Limbus	QH	14	NR	[50]
SCC	1	Limbus	Haflinger	12	NR	[49]
SCC	38	Limbus	13 Appaloosa, 8 Paint, 7 QH, 2 TB, and 8 NR	11.9 ± 5.3	NR	[45]
SCC	15	Limbus	Haflinger	2–16	NR	[40]
SCC	1	Limbus	Rocky Mountain Horse	9	NR	[42]
SCC	2	Limbus	Connemara	7–17	NR	[44]
CSI-SCC	10	Cornea	4 Appaloosa, 2 QH, 2 American Paint, 1 Pinto, and 1 TB	9–27.5	Chronic keratitis (5/10), immunomediated keratitis (1/10), and other corneo-limbal masses (3/10)	[11]
CSI-SCC	1	Cornea	Appaloosa	17	NR	[56]
HA	1	Limbus	TB	7	NR	[52]
HSA	1	Limbus	Arabian	15	NR	[53]
HSA	3	Limbus	Paint, Haflinger, and Clydesdale cross	8–20	NR	[54]
HSA	3	Cornea–limbus	Arabian, mule, and NR	9–21	NR	[28]

SCC, squamous-cell carcinoma; CSI-SCC, stromal invasive squamous-cell carcinoma; HSA, hemangiosarcoma; HA, hemangioma; QH, Quarter Horse; TB, Thoroughbred; NR, not reported.

## Data Availability

No new data were created or analyzed in this study.

## Abbreviations

The following abbreviations are used in this manuscript:

SCC	Squamous-Cell Carcinoma
c-SCC	Corneal Squamous-Cell Carcinoma
KCS	Kerato-Conjunctivitis Sicca
HA	Hemangioma
HSA	Hemangiosarcoma

## Appendix A

The clinical and histopathological data of cases from the archives of the authors are listed in Table A1, Table A2, Table A3, Table A4 and Table A5.

**Table A1:** Cases of canine corneo-limbal epithelial neoplasia.

Case	Breed	Sex	Age (Years)	Clinical History	Type of Sample	Site	Growth Pattern
1	Shih Tzu	M	6	Surgical conjunctival flap	Biopsy	C	Papilloma
2	Golden Retriever	FS	11	nd	Biopsy	CL	Papilloma
3	French Bouledogue	M	11	KCS, cyclosporine treatment	Biopsy	C	Papilloma + SCC in situ
4	Mixed breed	M	13	KCS	Biopsy + 2nd biopsy	C	Exophytic
5	Pug	FS	4	KCS, cyclosporine treatment	Biopsy	C	In situ
6	Bulldog	nd	7	nd	Biopsy	C	Exophytic
7	English Bulldog	M	5	KCS	Biopsy + 2nd biopsy	C	Exophytic
8	Bulldog	F	4	nd	Biopsy	C	Exophytic
9	English Bulldog	F	9	KCS, cyclosporine treatment	Biopsy	C	Exophytic, pseudoacinar
10	English Bulldog	FS	10	nd	Biopsy	C	Exophytic
11	CKCS	F	9	KCS, cyclosporine treatment	Biopsy + 2nd biopsy	C	Exophytic
12	CKCS	M	10	KCS, corneal ulcer, cyclosporine and tacrolimus treatment	Biopsy	C	Exophytic
13	Mixed breed	nd	nd	nd	Biopsy	C	Exophytic
14	Shih Tzu	MC	13	KCS, cyclosporine treatment	Biopsy	C	Exophytic
15	Pug	M	12	nd	Biopsy	C	Exophytic
16	Boston Terrier	F	10	KCS, corneal ulcer	Biopsy	C	Stromal invasive
17	Pug	FS	8	KCS, cyclosporine treatment, Leishmaniasis, corneal ulcer, surgical conjunctival flap	Biopsy	C	Infiltrative
18	French Bouledogue	M	6	Keratitis, chemotherapy for renal neoplasia (Palladia)	Biopsy	C	In situ
19	Pug	M	10	KCS	Biopsy	C	Infiltrative
20	English Bulldog	FS	8	KCS	Biopsy	C	Infiltrative
21	Shih Tzu	M	13	KCS	Biopsy	C	Stromal invasive
22	Shih Tzu	FS	11	KCS	Biopsy	C	Exophytic
23a	Mixed breed	M	nd	Leishmaniasis, granulomatous panophthalmitis	Eye globe OS	C	Exophytic
23b					Eye globe OD	C	Infiltrative
24	Mixed breed	M	5	nd	Biopsy	C	In situ
25	Shih Tzu	MC	12	KCS, cyclosporine treatment	Biopsy	C	Exophytic
26	English Bulldog	FS	10	nd	Biopsy	C	Exophytic
27	Pug	FS	9	KCS	Biopsy	C	In situ
28	Pug	MC	11	nd	Biopsy	C	Exophytic/infiltrative
29	French Bouledogue	M	5	nd	Biopsy	C	In situ
30	Pekingese	nd	13	nd	Eye globe	C	Exophytic
31	Chihuahua	M	15	nd	Biopsy	C	Exophytic
32	Pug	F	8	KCS, tacrolimus treatment	Biopsy	C	Exophytic
33	German Shepherd cross	FS	8	CSK	Eye globe	C	Exophytic
34	Pug	M	10	KCS	Biopsy	C	Exophytic
35	Shih Tzu	MC	11	KCS, tacrolimus treatment	Biopsy + 2nd biopsy	C	Exophytic to in situ to stromal invasive
36	English Bulldog	M	8	nd	Biopsy	C	Exophytic
37	Shih Tzu	FS	12	KCS, cyclosporine and tacrolimus treatment	Biopsy	C	Exophytic
38	English Bulldog	M	12	KCS, tacrolimus treatment	Biopsy	C	Exophytic
39	Mixed breed	FS	10	KCS	Biopsy	C	In situ
40	English Bulldog	FS	8	nd	Biopsy	C	Exophytic

M, intact male; MC; castrated male; F, intact female; FS, spayed female; KCS, keratoconjunctivitis sicca; CSK, chronic superficial keratitis; OS, left eye; OD, right eye; C, corneal; CL, corneo-limbal; nd, not determined.

**Table A2:** Cases of canine corneo-limbal vascular neoplasia.

Case	Breed	Sex	Age (Years)	Clinical History	Type of Sample	Site	Lesion
1	Abruzzes and Maremma Shepherd Dog cross	FS	6	nd	Eye globe	CL	HSA
2	German Shepherd Dog cross	F	10	KCS, previous corneal HSA contralateral eye	Biopsy	C	HA
3	Australian Shepherd	FS	8	nd	Biopsy	CL	HSA
4	Mixed breed	FS	5	nd	Biopsy	CL	HSA
5	Mixed breed	F	6	nd	Biopsy	CL	HSA
6	Mixed breed	FS	7	nd	Biopsy	CL	HSA
7	Australian Shepherd	FS	11	nd	Biopsy	CL	HA

F, intact female; FS, spayed female; KCS, keratoconjunctivitis sicca; C, corneal; CL, corneo-limbal; HA, hemangioma; HSA, hemangiosarcoma; nd, not determined.

**Table A3:** Cases of feline corneo-limbal epithelial neoplasia.

Case	Breed	Sex	Age (Years)	Clinical History	Type of Sample	Site	Growth Pattern
1	DSH	F	10	nd	Eye globe	C	Stromal invasive
2	DSH	MC	5	nd	Biopsy	CL	Infiltrative
3	DSH	FS	13	nd	Biopsy	CL	Exophytic
4	DSH	M	5	Ocular trauma , glaucoma	Eye globe	CL	Exophytic
5	DSH	F	10	nd	Biopsy + eye globe	C	Stromal invasive
6	DSH	MC	4	symblepharon	Eye globe	CL	Infiltrative, pseudoacinar
7	DSH	nd	7	FHV1, symblepharon	Biopsy	C	Infiltrative, pseudoacinar
8	DSH	FS	18	Chronic keratitis, symblepharon	Eye globe	CL	Exophytic/infiltrative, pseudoacinar
9	DSH	MC	10	Eosinophilic keratitis	Eye globe	C	Infiltrative
10	DSH	MC	8	nd	Biopsy + eye globe	CL	Exophytic
11	DSH	nd		symblepharon	Biopsy	CL	Infiltrative
12	DSH	MC	10	symblepharon	Biopsy + eye globe	CL	Exophytic
13	DSH	MC	8	nd	Eye globe	CL	Infiltrative
14	DSH	MC	5	nd	Biopsy	C	Stromal invasive, pseudoacinar

DSH, Domestic Short Haired; M, intact male; MC, castrated male; F, intact female; FS, spayed female; FHV, Feline Herpesvirus; C, corneal; CL, corneo-limbal; nd, not determined.

**Table A4:** Cases of feline corneo-limbal vascular neoplasia.

Case	Breed	Sex	Age (Years)	Clinical History	Type of Sample	Site	Lesion
1	DSH	F	10	Symblepharon	Biopsy	C	HA
2	DSH	FS	14	nd	Biopsy	C	HSA
3	DSH	MC	9	Palpebral agenesia, trichiasis	Biopsy	C	HA
4	DSH	FS	10	nd	Eye globe	C	HSA
5	DSH	M	13	nd	Biopsy	C	HSA
6	DSH	MC	6	nd	Biopsy	C	HSA
7	DSH	MC	12	FHV1, symblepharon	Eye globe	C	HA
8	DSH	MC	9	Symblepharon	Eye globe	C	HSA
9	DSH	M	3	Symblepharon	Biopsy	C	HA
10	DSH	MC	8	Corneal ulcer	Eye globe	C	HA
11	DSH	F	nd	nd	Eye globe	C	HSA
12	DSH	FS	12	FHV1	Eye globe	CL	HA
13	DSH	MC	12	Corneal perforation	Eye globe	C	HSA
14	DSH	MC	8	Symblepharon	Biopsy	C	HA + SCC
15	DSH	M	8	nd	Biopsy	C	HA

DSH, Domestic Short Haired; M, intact male; MC, castrated male; F, intact female; FS, spayed female; FHV, Feline Herpesvirus; C, corneal; CL, corneo-limbal; HA, hemangioma; HSA, hemangiosarcoma; nd, not determined.

**Table A5:** Cases of equine corneo-limbal epithelial neoplasia.

Case	Breed	Sex	Age (Years)	Type of Sample	Site	Growth Pattern
1	Arabian	MC	18	Biopsy	CL	Infiltrative
2	Mérens	F	16	Biopsy + eye globe	C	Stromal invasive
3	Appaloosa (pony)	MC	16	Biopsy	C	Infiltrative
4	Maremmano	MC	29	Biopsy	CL	Exophytic
5	Pony	F	15	Biopsy	CL	Exophytic
6	Welsh Pony	F	20	Biopsy	CL	Stromal invasive
7	Paint	MC	20	Biopsy	CL	Infiltrative
8	Argentine Criollo	MC	21	Biopsy	CL	Exophytic
9	Argentine Criollo	F	17	Biopsy	CL	Infiltrative
10	Argentine Criollo	MC	19	Biopsy	C	In situ
11	nd	F	15	Biopsy	CL	Infiltrative
12	nd	F	9	Biopsy	CL	Exophytic
13	nd	F	20	Biopsy	C	Exophytic
14a	Haflinger	MC	16	Biopsy OD	CL	Exophytic
14b				Biopsy OS	CL	In situ
15	KWPN	F	14	Biopsy	CL	Infiltrative
16	Arabian	F	22	Eye globe	CL	Infiltrative
17	Appaloosa	MC	18	Biopsy	C	In situ
18	Haflinger–Appaloosa cross	F	15	Biopsy	CL	Infiltrative

MC, gelding; F, mare; OD, right eye; OS, left eye; C, corneal; CL, corneo-limbal; nd, not determined.
